# Explainable Remaining Useful Life Prediction of Air Circuit Breakers via Physics-Informed Electro-Mechanical Feature Fusion

**DOI:** 10.3390/s26185802

**Published:** 2026-09-13

**Authors:** Jiaqing Zhou, Wei Chen, Jintao Chen, Xinhao Chen

**Affiliations:** 1 Zhejiang Key Laboratory of Smart Low-Voltage Apparatus and New Energy Application, Wenzhou University, Wenzhou 325035, China; 25451251032@stu.wzu.edu.cn (J.Z.);; 2 School of Mechanical Engineering, Zhejiang University, Hangzhou 310007, China; 3 Zhejiang Tengen Electric Co., Ltd., Wenzhou 325600, China

**Keywords:** air circuit breaker, remaining useful life, electromechanical feature fusion, random forest, asymmetric penalty score, condition-based maintenance, permutation importance analysis

## Abstract

Accurate remaining useful life (RUL) prediction of air circuit breakers (ACBs) is crucial for condition-based maintenance. However, existing data-driven prognostic methods suffer from electromechanical feature fragmentation, cross-device domain shifts, and the inability to penalize safety-critical late predictions. This study proposes an explainable RUL prediction framework via physics-informed feature fusion. Through full-lifecycle monitoring, novel indicators, including the electromechanical coupled degradation index (EMCDI) and the contact spring over-travel consumption rate (CSOCR), are introduced to decode interactive degradation cycles. To eliminate the interferences of initial manufacturing tolerances, a phase-decoupled normalization strategy empowers a random forest (RF) model to achieve cross-device transferability in a two-device proof-of-concept experiment, requiring only 50 initial operations for target calibration. Additionally, a safety-oriented asymmetric penalty score (APS) is integrated into the evaluation framework to explicitly penalize hazardous life overestimations. Experimental results demonstrate a full-lifecycle *R*^2^ of 0.9936 and a mean absolute error (MAE) of 38.5136. While the early-stage *R*^2^ of 0.6880 objectively reflects the statistical flatness of the equipment’s healthy plateau, the framework maintains robust tracking capabilities across the entire lifespan, surpassing mainstream deep learning algorithms such as CNN, MLP, and LSTM. The proposed method consistently achieves a conservative, risk-averse predictive distribution for industrial reliability. Finally, model-level permutation importance analysis confirms that the RF model prioritizes physics-informed indicators rather than relying on spurious curve fitting.

## 1. Introduction

Air circuit breakers (ACBs) serve as critical protective components in low-voltage distribution networks. Their primary function is to safely isolate fault conditions by interrupting massive short-circuit currents, thereby safeguarding electrical infrastructures from catastrophic damage. The reliable operation of circuit breakers depends heavily on standard operation and maintenance procedures. Traditionally, asset management has relied on time-based maintenance (TBM) strategies. However, studies emphasize that conventional TBM often results in inefficient resource allocation and fails to detect sudden equipment degradation [1,2]. To improve system reliability, researchers such as Zhong et al. and Leone et al. have proposed data-driven approaches to determine maintenance priorities and assess prognostic similarities [3,4]. Consequently, there is an urgent industry shift toward condition-based maintenance (CBM) and predictive maintenance (PdM). Accurately estimating the remaining useful life (RUL) of ACBs is essential for ensuring the resilience and safety of modern power grids.

The performance degradation of an ACB is a highly complex, multiphysical process characterized by mechanical wear and electrical contact ablation. In the mechanical domain, researchers frequently analyze vibration signals generated during opening and closing operations. For instance, Yan et al. utilized a multilayer perceptron (MLP) for vibration-based fault diagnosis [5], while Qu et al. optimized stochastic configuration networks (SCNs) for similar tasks [6]. To address the scarcity of fault data, Yang et al. proposed a vibration signal augmentation method based on generative adversarial networks (GANs) [7], and Yao et al. combined fractal theory with probabilistic neural networks for miniature circuit breakers [8]. Furthermore, Han et al. developed structural optimization strategies to match the suction characteristics of the electromagnetic operating mechanism [9]. In the electrical domain, Kang et al. investigated the degradation of polymer bonding structures in insulation materials under thermal stress [10], and Zhang et al. employed bidirectional long short-term memory (BiLSTM) networks to detect the state of low-voltage switch trip circuits [11].

With the rapid development of artificial intelligence, data-driven deep learning models have increasingly dominated switchgear prognostics. Alsumaidaee et al. reviewed deep learning applications for medium-voltage switchgear fault detection [12], later implementing a hybrid 1D-CNN-LSTM model to classify faults in the time and frequency domains [10]. For quantitative life prediction, Zhou et al. developed a bidirectional gated recurrent unit (BiGRU) model for molded case circuit breakers [13], while Ouyang et al. applied a Savitzky–Golay smoothed LSTM network to predict the residual electrical life of AC circuit breakers [14]. Wu et al. introduced wavelet-enhanced dual-tree residual networks for operating mechanisms [15]. Additionally, Sun et al. extensively explored advanced neural networks, including self-attention BiLSTM frameworks [16], improved AlexNet architectures [17], and multichannel convolutional auto-encoders (MCCAE) [18,19] to extract degradation-sensitive vibration features. To quantify the uncertainty inherent in long-term degradation, Cathignol et al. and Zhuang et al. introduced Bayesian inference and hierarchical deep learning approaches [20,21]. Similarly, Zhou et al. and Zhao et al. utilized functional principal component analysis (FPCA) to extract degradation trends in high-voltage and vacuum circuit breakers [18,22]. Beyond predictive accuracy, recent data-driven engineering studies have increasingly coupled model prediction with global and local explainability analyses and physics-based verification, showing that physically interpretable machine learning frameworks can reveal the factors and process stages responsible for individual predictions rather than treating prediction accuracy alone as evidence of mechanistic understanding [23,24].

Despite the proliferation of these intelligent diagnostic frameworks, the practical deployment of RUL prediction for ACBs in industrial environments is still severely impeded by three fundamental bottlenecks: (1) Physical fragmentation: Most existing studies evaluate ACB health by decoupling the electrical and mechanical domains. For instance, models relying solely on vibration signals [5,7] or pure electrical features [14] fail to capture the interactive mechanical–jamming–electrical vicious cycle that governs mid-to-late stage degradation. Although multisource signal fusion has been recently explored by Zhou et al. [25] and Wang et al. [26], a comprehensive framework fusing physics-informed electromechanical features specifically for ACB RUL tracking remains absent. Related physics-informed data-driven frameworks in other complex engineering processes have further shown that interpretability can be strengthened when relationships identified by predictive models and explainable artificial intelligence are independently corroborated through multiphysical simulation, thereby distinguishing mere data-driven statistical correlations from genuine degradation mechanisms supported by complementary physical evidence [27,28]. (2) Small-sample generalization disaster: As noted by Winkel et al., acquiring extensive run-to-failure datasets for industrial switchgear is prohibitively expensive [29]. Highly parameterized models tend to overfit the specific mechanical tolerances of the training equipment. While Wang et al. proposed a prior knowledge-constrained wavelet network to address few-shot fault diagnosis, mitigating the cross-device domain shift in continuous RUL regression tasks remains a critical challenge [30]. (3) Misalignment of evaluation metrics: Current prognostic studies ubiquitously employ symmetric evaluation metrics, such as mean absolute error (MAE) or root mean square error (RMSE). In safety-critical power systems, prediction errors possess highly asymmetric consequences. Overestimating the RUL leaves a severely degraded ACB in service, directly risking thermal runaway and widespread outages, whereas underestimating only prompts premature maintenance.

To bridge these gaps, this study proposes a novel explainable RUL prediction framework for ACBs via physics-informed electro-mechanical feature fusion. By decoding the interactive deterioration mechanisms and introducing a robust random forest (RF) algorithm driven by phase-decoupled baseline normalization, this study achieves high-precision, cross-device RUL tracking. Furthermore, an asymmetric penalty score (APS) is integrated into the evaluation framework to explicitly penalize hazardous late predictions, strictly satisfying the safety demands of industrial engineering applications.

## 2. Experimental Setup and Degradation Data Acquisition

### 2.1. Experimental Setup

**Figure 1:**
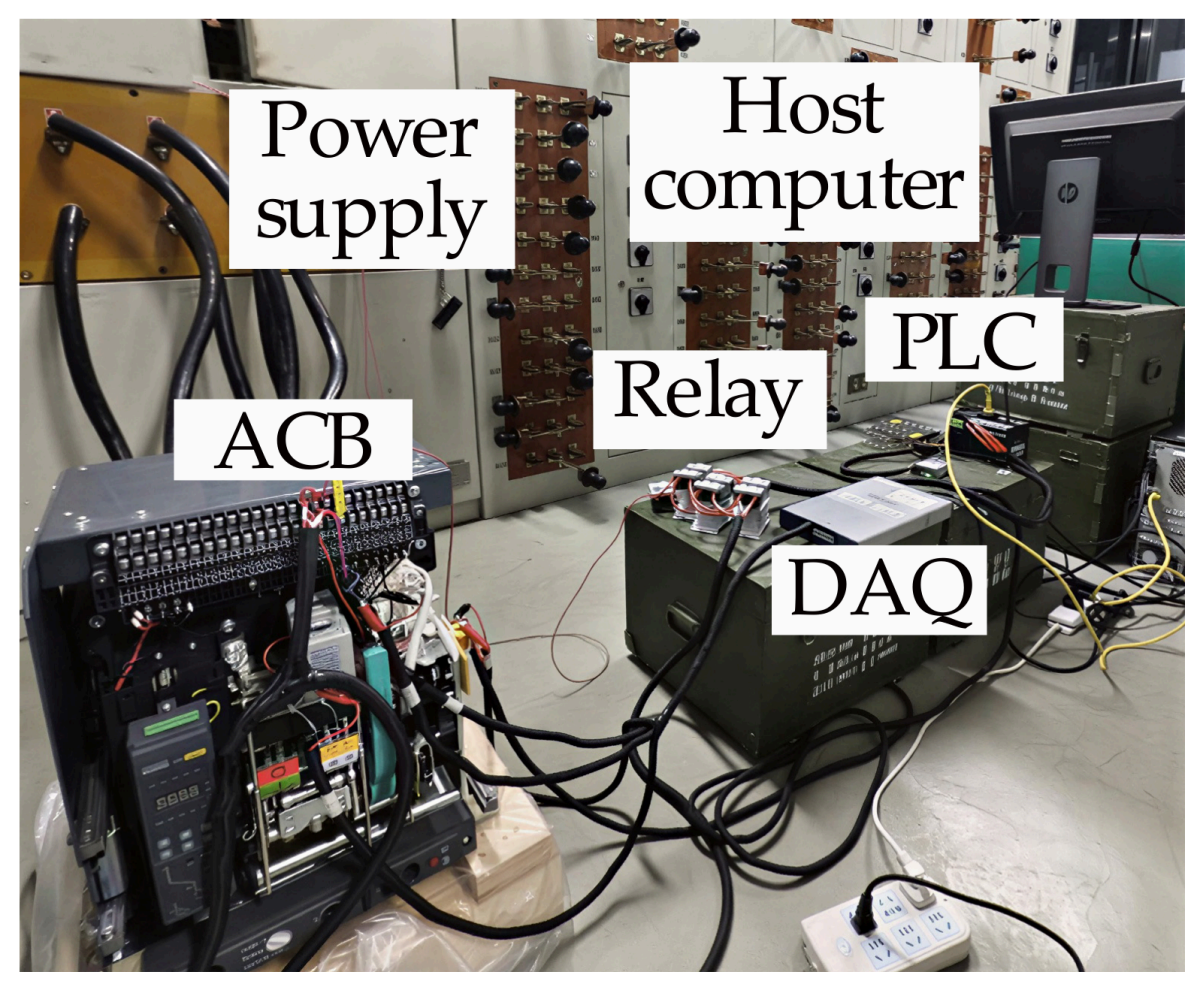
Experimental setup of the electrical life test.

To accurately predict the remaining service life of the ACBs, a customized multisignal synchronous test platform, shown in Figure 1, was built to capture the full degradation process of the ACB from a factory-new state to eventual failure, obtaining electrical and mechanical data spanning their entire lifecycles. The electric life test platform primarily consists of a power supply system, a multichannel data acquisition (DAQ) module, a programmable logic controller (PLC), a relay group, a host computer and the test objects. LabVIEW 2024 Q1 software was utilized on the host computer to compile the control and synchronous data acquisition programs for automated electrical life testing. The opening and closing of the ACBs were controlled by the PLC and the relay group. An NI USB-6366 data acquisition module was employed to synchronously sample the electrical and mechanical signals, with the sampling frequency set to 50 kHz.

### 2.2. Data Acquisition

The test objects comprised two ACBs with a rated voltage of 400 V AC and a rated current of 2000 A, both of which utilize silver-tungsten contacts and a spring energy storage operating mechanism. The electrical life tests were carried out under power frequency AC load conditions, and the operational procedures strictly adhered to the GB/T 14048.2-2020 standard [31]. The switching frequency of the ACBs was set at 20 operations per hour, with a 3-min interval between consecutive cycles. This interval ensured sufficient heat dissipation of the test objects and eliminated additional thermal effects caused by continuous arcing, thereby accurately simulating industrial operating conditions. The tests continued until the ACBs failed to close and conduct current in any phase. Testing was suspended every 50 switching cycles to manually check the contact wear and over-travel, recording the degradation states of the ACBs.

To comprehensively monitor the electromechanical coupled degradation states of the ACBs, the acquired data encompass both electrical and mechanical signals. The voltage and current waveforms of the main circuit, along with the current waveforms of the opening and closing coils, were captured using voltage and current sensors with measurement errors of less than 0.5%.

**Figure 2:**
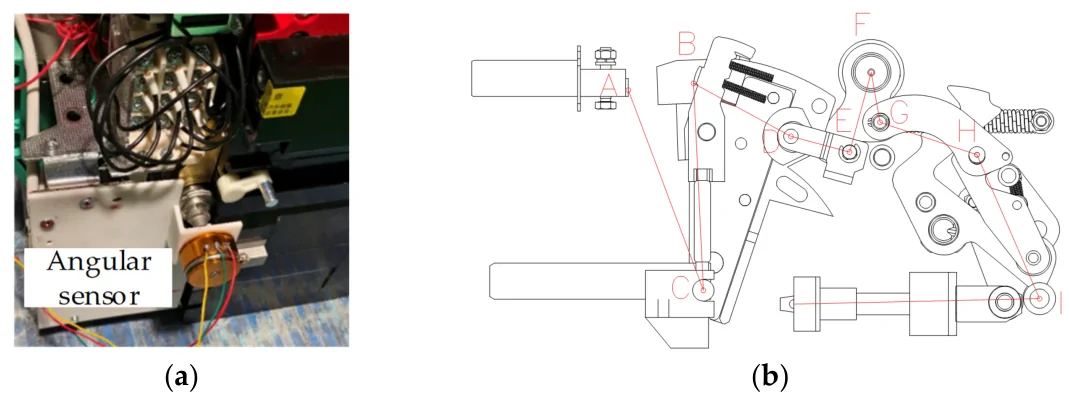
Schematic diagram of mechanical signal measurement. (**a**) Installation of the angular sensor; (**b**) Simplified schematic diagram of operating mechanism for kinematic analysis.

A conductive plastic angular sensor with a linearity of 0.1% was coaxially installed at the spindle end of the operating mechanism to continuously measure the spindle’s angular displacement throughout the entire switching action, as shown in Figure 2a. The ACB operating mechanism can be divided into two parts: the driving mechanism and the moving contact mechanism. Both can be modeled as equivalent four-bar linkage systems, as depicted in Figure 2b. A deterministic geometric constraint relationship exists between the rotation angles of the spindle (Point F in Figure 2b) and the moving contacts (Point B in Figure 2b). Through a kinematic analysis of the operating mechanism, the angular displacement of the moving contacts can be analytically derived from that of the spindle.

## 3. Data Preprocessing and Multidimensional Feature Engineering

After obtaining the multisource synchronous monitoring data throughout the full lifecycle of the ACBs, identifying features that accurately map the internal degradation mechanism with high generalization ability becomes crucial for achieving high-precision RUL prediction. This section first preprocesses the original signals. It then constructs a four-dimensional feature matrix based on physical degradation mechanisms and finally proposes a feature benchmarking strategy based on phase-angle decoupling to eliminate data distribution shifts in cross-device prediction.

### 3.1. Original Signal Preprocessing and Action Interval Extraction

A significant amount of redundant static data was present in the continuous multi-second data records of a single test, so it is necessary to accurately extract the data segments of the closing, normal steady-state conduction, and opening processes. The closing process is defined from the closing coil trigger time $t_{0,close}$ to the time $t_{end,close}$ when the spindle’s angular displacement reaches a steady state. The opening process is defined from the opening coil trigger time $t_{0,open}$ to the time $t_{end,open}$ when the spindle’s angular displacement stabilizes. This segmentation effectively reduces the computational burden and ensures the precise alignment of multisource waveforms in the time dimension.

**Figure 3:**
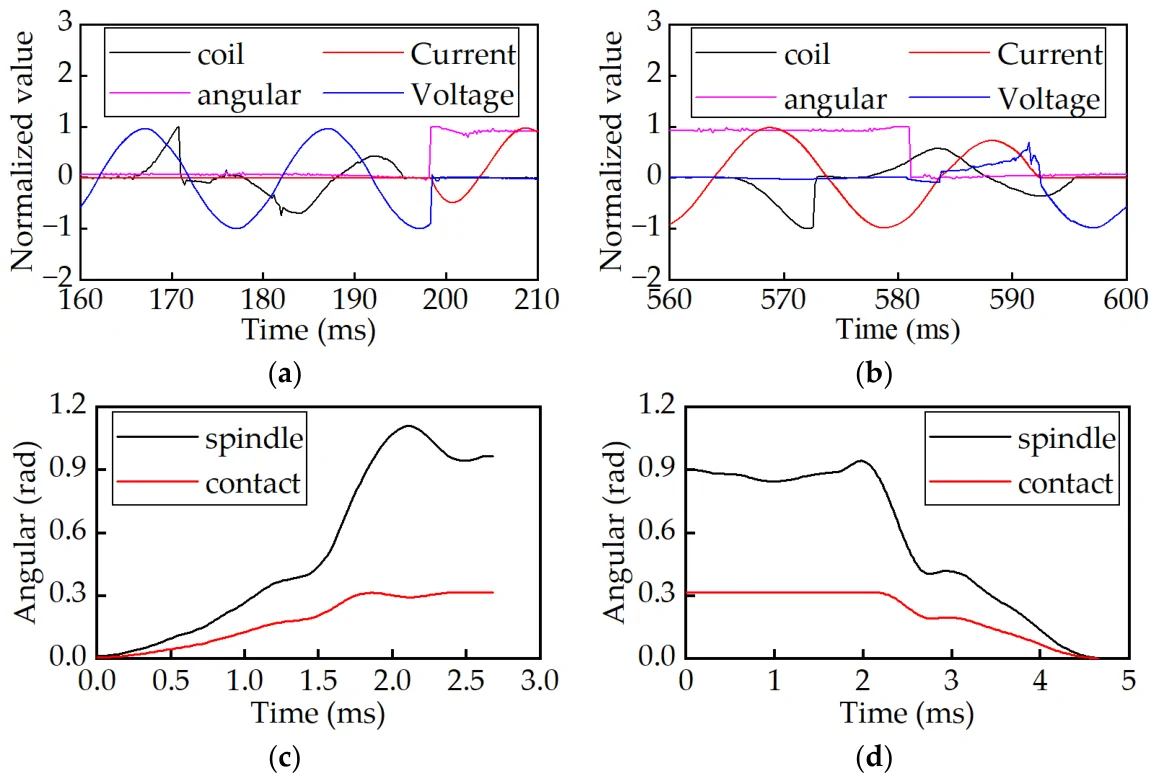
Normalized values of closing or opening exciting coil current, angular sensor output voltage, main circuit current, and main circuit voltage. (**a**) Normalized values during closing process. (**b**) Normalized values during opening process. (**c**) Angular displacements of the spindle and moving contacts during closing process. (**d**) Angular displacements of the spindle and moving contacts during opening process.

Due to electromagnetic interference in industrial environments and the inherent high-frequency thermal noise of the sensors, inevitable spikes appeared in the originally acquired waveforms. To retain the true phase characteristics of the mechanical transient response, the high-frequency sampled mechanical angular signals were smoothed using a zero-phase low-pass filter, which effectively removes high-frequency noise and avoids phase delays. Examples of the electrical and mechanical signals during the closing and opening processes are presented in Figure 3.

### 3.2. Feature Extraction and Mechanism Mapping

The aging of ACBs is a complex multiphysical coupling process involving arc ablation, mechanical wear, and material fatigue. Characteristics from a single physical dimension can only reflect localized health states. Therefore, this study constructs a comprehensive degradation feature matrix across four dimensions: electrical, mechanical, electromechanical coupling, and time-series accumulation, as summarized in Table 1. This matrix not only covers the direct electrical wear of the contact system but also captures the indirect fatigue of the operating mechanism and the system-level response hysteresis.

#### 3.2.1. Electrical Features

By analyzing waveform variations during switching operations, an algorithm combining threshold screening and waveform distortion rates was employed to accurately locate the start moment $t_{\mathrm{arc}\_\mathrm{start}}$ and end moment $t_{\mathrm{arc}\_\mathrm{end}}$ of the closing and opening processes. The single arc energy $W_{a}$ is defined as the integral of transient main-circuit voltage $u_{m}\left(t\right)$ and current $i_{m}\left(t\right)$:

(1) $$W_{\mathrm{arc}}=\int_{t_{\mathrm{arc}\_\mathrm{start}}}^{t_{\mathrm{arc}\_\mathrm{end}}}u_{m}\left(t\right)\cdot i_{m}\left(t\right)dt$$

To comprehensively describe the arc characteristics, the arcing time $T_{\mathrm{arc}}$, switching phase angle $\phi_{\mathrm{switch}}$, initial voltage slope of the opening arc $du_{\mathrm{arc}}/dt$, transient contact resistance $R_{\mathrm{constant}}$, and coil-to-arc response delay $\Delta T_{\mathrm{delay}}$ were extracted. Among these electrical features, $du_{\mathrm{arc}}/dt$ serves as an electrical surrogate for the moving contact’s separation speed. In a healthy ACB, the arc is rapidly stretched and squeezed into the arc-extinguishing grid, causing the arc voltage to rise rapidly. However, as contact ablation, surface roughness, and metal vapor concentration in the arc chamber increase, the resistance encountered by the arc entering the grid also increases, significantly decreasing the voltage build-up rate. Equation (2) defines the average rate of voltage changes within the initial time window after arcing.

(2) $$\frac{du_{\mathrm{arc}}}{dt}=\frac{u_{m}\left(t_{\mathrm{arc}\_\mathrm{start}}+\Delta t_{\mathrm{init}}\right)-u_{m}\left(t_{\mathrm{arc}\_\mathrm{start}}\right)}{\Delta t_{\mathrm{init}}}$$

where $\Delta t_{\mathrm{init}}$ is the initial time window following arc ignition, chosen as 0.2 ms for slope fitting, and $t_{\mathrm{arc}\_\mathrm{start}}$ is the arc occurrence time.

To capture resistance characteristics that genuinely reflect the deterioration state of the contact surfaces, this study avoided calculations during the arcing stage or intervals of violent transient fluctuations period. Instead, a short-term stable conduction interval preceding the physical separation of the contacts was selected. Based on the transient voltage drop and the main circuit current, the transient contact resistance $R_{\mathrm{constant}}$ is defined as the averaged integral over this specific time window.

(3) $$R_{\mathrm{contact}}=\frac{1}{\Delta T_{\mathrm{delay}}}\int_{t_{\mathrm{coil}}}^{t_{\mathrm{arc}\_\mathrm{start}}}|\frac{u_{m}(t)}{i_{m}(t)}|dt$$

where $t_{\mathrm{coil}}$ is the coil trigger time.

In three-phase ACBs, the arc energy across the three phases is typically unbalanced due to the randomness of the breaking phase angles and minor differences in the initial wear states of the phase contacts. This imbalance not only causes asymmetric ablation of the contacts but also generates asymmetric electromagnetic repulsion and thermal stresses, which in turn impose additional eccentric torque on the mechanical spindle during operation. Therefore, the three-phase energy asymmetry index (TPEAI) was introduced to reflect the mechanical jamming tendencies induced by uneven three-phase erosion. To eliminate the influence of absolute energy amplitude fluctuations under varying current loads, the TPEAI utilizes relative dispersion for evaluation. It is mathematically defined as the unitary deviation of the three-phase arc energy relative to its mean value:

(4) $$\mathrm{TPEAI}=\frac{1}{{\bar{W}}_{\mathrm{arc}}}\sqrt{\frac{1}{3}\sum_{x\in \left\{A,B,C\right\}}{\left(W_{\mathrm{arc},x}-{\bar{W}}_{\mathrm{arc}}\right)}^{2}}$$

where ${\bar{W}}_{\mathrm{arc}}$ is the average arc energy across the three phases, and $W_{\mathrm{arc},x}$ is the arc energy of phase *x*.

#### 3.2.2. Mechanical Features

Mechanical degradation in ACBs primarily stems from energy-storage spring fatigue, the deterioration and drying of lubricating grease, and irreversible wear within the four-bar linkage transmission system. To comprehensively quantify these physical deteriorations, five features were extracted from the high-frequency angular sensor signals: average opening angular velocity $\bar{\omega }$, contact initial separation angular velocity $\omega_{\mathrm{sep}}$, total contact angular stroke $\theta_{\mathrm{total}}$, contact over-travel loss angle $\Delta \theta_{\mathrm{loss}}$, and mechanical motion time $T_{\mathrm{motion}}$.

As the operating mechanism accumulates fatigue, increased frictional resistance within the bearings directly prolongs $T_{\mathrm{motion}}$. Based on this time window and the continuous angular displacement trajectory $\theta \left(t\right)$ captured by the sensor, $\bar{\omega }$ is calculated to evaluate macroscopic transmission efficiency and the overall attenuation of the spring force:

(5) $$\bar{\omega }=\frac{\theta \left(t_{\mathrm{move}\_\mathrm{end}}\right)-\theta \left(t_{\mathrm{move}\_\mathrm{start}}\right)}{T_{\mathrm{motion}}}$$

where $\theta \left(t_{\mathrm{move}\_\mathrm{end}}\right)$ and $\theta \left(t_{\mathrm{move}\_\mathrm{start}}\right)$ are the angular positions at the start and end times, respectively. While $\bar{\omega }$ reflects the global kinematic state, $\omega_{\mathrm{sep}}$ is critical for evaluating the instantaneous arc-extinguishing capability. A lower $\omega_{\mathrm{sep}}$ indicates a slower initial arc-stretching rate, which directly exacerbates thermal stresses on the contacts. It is defined as the instantaneous derivative of the angular displacement at the exact moment of moving contact separation:

(6) $$\omega_{\mathrm{sep}}=\frac{d\theta \left(t_{sep}\right)}{dt}$$

where $t_{sep}$ denotes the moment of physical contact separation, which is considered equivalent to the arc initiation time. From a geometric perspective, $\theta_{\mathrm{total}}$ is intrinsically constrained by the rigid mechanical limiters of the four-bar system, serving as a macroscopic structural boundary. Conversely, $\Delta \theta_{\mathrm{loss}}$ serves as a direct spatial mapping of microscopic material ablation.

Through these mathematical definitions, abstract mechanical degradation phenomena, such as mechanism jamming, spring fatigue, and physical contact mass loss, are strictly mapped into quantifiable geometric and kinematic feature vectors, laying a solid data foundation for subsequent electromechanical coupling analyses.

#### 3.2.3. Electromechanical Coupling Features

An electromechanical vicious cycle emerges during the mid-to-late service stages of ACBs. Mechanism jamming slows the opening speed, which prolongs the arc combustion time and generates severe thermal stresses. These thermal stresses accelerate metal splashing on the contact surfaces and contaminate the surrounding mechanical structures, which, in turn, further aggravates the jamming of the mechanism.

To quantify this interactive degradation effect, the electromechanical coupled degradation index (EMCDI) is defined as the nonlinear ratio of the total three-phase arc energy to the contact initial separation angular velocity $\omega_{\mathrm{sep}}$, formulated as follows:

(7) $$\mathrm{EMCDI}=\frac{\sum_{x\in \left\{A,B,C\right\}}W_{\mathrm{arc},x}}{\omega_{\mathrm{sep}}}$$

To guarantee sufficient contact pressure even after substantial ablation, the moving contact assemblies of ACBs are typically designed with an over-travel allowance. As the silver-tungsten alloy contacts thin due to the repeated interruption of high-temperature arcs, the mechanical over-travel angle in the closed state gradually decreases. Based on continuous spindle angular displacement monitoring, the contact spring over-travel consumption rate (CSOCR) is defined as:

(8) $$\mathrm{CSOCR}=\frac{\Delta \theta_{\mathrm{loss}}}{\sum_{j=1}^{k}W_{\mathrm{arc},j}}$$

where $W_{\mathrm{arc},j}$ is the arc energy of the *j*th operation.

#### 3.2.4. Time-Series Accumulation Features

When processing cross-sectional data, machine learning algorithms (such as random forest, support vector machine, etc.) usually treat the feature vector of each switching operation as an independent sample, lacking awareness of historical states along the temporal axis. However, the physical aging of ACBs is an irreversible, long-term cumulative damage process. Relying solely on the transient characteristics of single operations renders the model vulnerable to interferences from random breaking phase angles, load fluctuations, and measurement noise, resulting in unreasonable oscillations in the predicted RUL trajectories.

To mitigate this issue, cumulative and sliding statistical features in the time-series dimension were explicitly incorporated into the feature matrix, including the cumulative operation count $N_{\mathrm{count}}$, cumulative arc energy $W_{\mathrm{arc}}\left(k\right)$, cumulative cut-off joule integral $\sum I^{2}t$, moving average of response delay $\mu_{\Delta T}$, and moving variance of response delay $\sigma_{\Delta T}^{2}$. The core objective is to inject global degradation trends and localized instability patterns into the data-driven model, effectively overcoming the memoryless defect of conventional algorithms.

In actual operation, the single-operation electromechanical response delay $\Delta T_{\mathrm{delay}}$ is highly susceptible to ambient temperature variations and fluctuations in the control coil terminal voltage. To extract the genuine mechanism degradation baseline, a sliding window is applied to calculate its moving average as follows:

(9) $$\mu_{\Delta T}\left(k\right)=\frac{1}{L}\sum_{j=k-L+1}^{k}\Delta T_{\mathrm{delay}}\left(j\right)$$

where *L* is the sliding window size, representing the number of consecutive operations (empirically set to 10). The moving average of response delay $\mu_{\Delta T}$ effectively filters out high-frequency transient noise, extracting the low-frequency degradation trend of the operating mechanism caused by progressive grease drying and steadily increasing bearing friction. This enables the model to stably track the mechanism jamming process.

Utilizing the same time sliding window, the moving variance of response delay $\sigma_{\Delta T}^{2}$ is calculated via Equation (10), aiming to compensate for the delayed response of global cumulative metrics to sudden late-stage mutations.

(10) $$\sigma_{\Delta T}^{2}\left(k\right)=\frac{1}{L-1}\sum_{j=k-L+1}^{k}{\left(\Delta T_{\mathrm{delay}}\left(j\right)-\mu_{\Delta T}\left(k\right)\right)}^{2}$$

As the ACB approaches its end-of-life, severe wear of mechanical components and increased kinematic clearances make the mechanism highly susceptible to stick-slip phenomena. Consequently, $\sigma_{\Delta T}^{2}$ spikes sharply, serving as a strong precursor signal that indicates an impending mechanical jamming or conduction failure.

### 3.3. Phase-Decoupled Baseline Normalization

Although absolute physical features contain rich degradation information, directly feeding them into prediction models causes severe data distribution shifts in cross-device transfer scenarios. This primarily stems from two factors: first, the initial manufacturing tolerances and assembly variances among different ACBs; Second, the randomness of the breaking phase angle, which causes sharp fluctuation of the instantaneous amplitude of the cut-off current. Consequently, arc energy and related characteristics become submerged in phase-angle noise.

To eliminate the above interference, this study proposes a phase-decoupled feature normalization strategy, shifting the model’s learning objective from fitting absolute physical quantities to tracking relative degradation trends. Leveraging the half-wave symmetry of AC currents, the switching phase angle is mapped to the [0~π] interval and equally partitioned into 12 sub-intervals $\Phi_{m}$ (indexed by m=1,2,…,12). To ensure normalization robustness, if a specific phase sub-interval remains empty during the initial 50 calibration operations, its baseline value is linearly interpolated from adjacent non-empty sub-intervals. The feature reference values for each sub-interval are established using the health-state data from the first 50 operations.

(11) $$X_{\mathrm{base}}\left(\Phi_{m}\right)=\frac{1}{N_{m}}\sum_{k\in \Phi_{m},k\leq 50}X\left(k\right)$$

where $X_{\mathrm{base}}\left(\Phi_{m}\right)$ is the baseline value of feature *X* within the *m*th phase-angle sub-interval, $N_{m}$ is the number of operations falling into $\Phi_{m}$ in the first 50 cycles, and $X\left(k\right)$ is the original feature value of the *k*th operation. For the *k*th operation occurring in the later service stages, amplitude-class features (such as energy and time metrics) are normalized via a ratio against the specific baseline value matched by the switching phase angle $\phi_{\mathrm{switch}}$ of the separation:

(12) $$X_{\mathrm{norm}}\left(k\right)=\frac{X\left(k\right)}{X_{\mathrm{base}}\left(\Phi_{m}\right)}$$

The proposed phase-decoupled baseline normalization study forces the model to ignore absolute initial offsets and instead learn the relative deterioration rate, thereby improving feature monotonicity and cross-device generalization capabilities.

## 4. RUL Prediction Framework Based on Random Forest Model

Since the multisource features of ACBs exhibit high dimensionality, strong non-linearity, and intense localized fluctuation, this study first employs the Spearman rank correlation coefficient to reduce dimensionality and optimize feature selection. Subsequently, an RUL regression model based on the RF algorithm is constructed. Finally, a rigorous verification strategy, which encompasses both single-device closed-loop validation and cross-device transfer generalization, is detailed to comprehensively evaluate the model’s feasibility for industrial application.

### 4.1. Feature Evaluation and Selection Based on Spearman Rank Correlation

**Figure 4:**
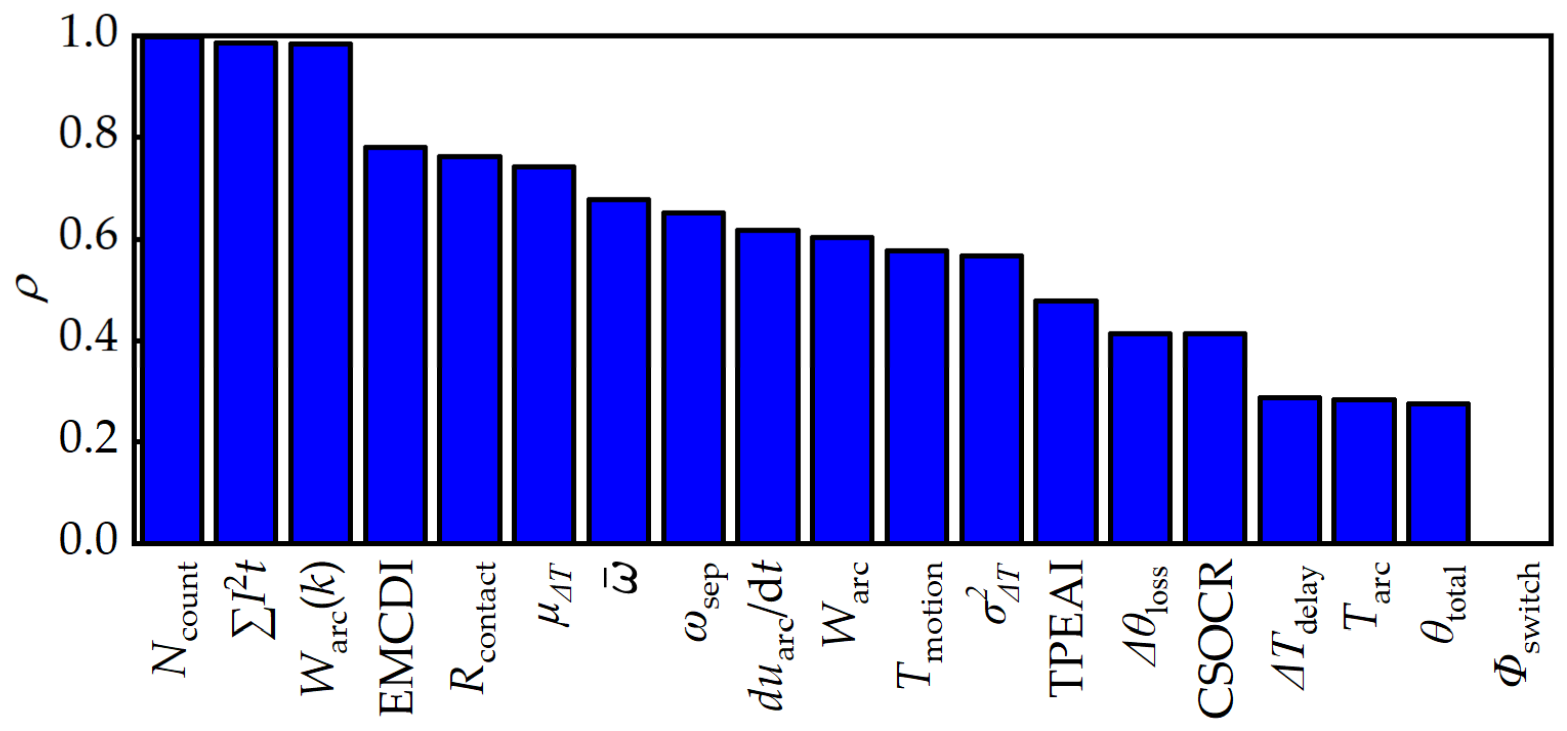
Spearman rank correlation of multi-dimensional features.

Before feeding the multidimensional feature matrix constructed in Section 3 into the prediction model, feature dimensionality reduction is imperative. This process eliminates redundant variables, reduces model complexity and mitigates the risks of statistical inference distortion and overfitting caused by high-dimensional sparsity. Considering that the physical degradation process of ACBs (such as contact thinning and mechanism jamming) exhibits a non-linear monotonic deterioration trend, the Pearson correlation coefficient, which is traditionally used to measure linear relationships, is inadequate. Therefore, this study employs the Spearman rank correlation coefficient ρ to quantify the monotonic correlation between the individual features and the true degradation timeline.

(13) $$\rho =\frac{\sum_{i=1}^{n}\left(R\left(X_{i}\right)-{\bar{R}}_{X}\right)\left(R\left(Y_{i}\right)-{\bar{R}}_{Y}\right)}{\sqrt{\sum_{i=1}^{n}{\left(R\left(X_{i}\right)-{\bar{R}}_{X}\right)}^{2}\cdot \sum_{i=1}^{n}{\left(R\left(Y_{i}\right)-{\bar{R}}_{Y}\right)}^{2}}}$$

where $R\left(X_{i}\right)$ and $R\left(Y_{i}\right)$ are the ranks of the originally converted values, ${\bar{R}}_{X}$ and ${\bar{R}}_{Y}$ are the mean values of their ranks. Based on the preprocessed valid samples and the multidimensional feature set, the feature matrix and lifetime label matrix were constructed to calculate the coefficient ρ of each feature. The Spearman rank correlation results are illustrated in Figure 4.

As depicted in Figure 4, the extracted features exhibit varying degrees of monotonic correlation with the degradation timeline. The time-series accumulation features, specifically the cumulative operation count $N_{\mathrm{count}}$, cumulative arc energy $W_{\mathrm{arc}}\left(k\right)$, and cumulative cut-off joule integral $\sum I^{2}t$, demonstrate the highest Spearman rank correlation coefficients, approaching 1.0. It is critical to emphasize that this near-perfect correlation is mathematically predetermined by their strictly additive physical definitions. These cumulative indices were deliberately incorporated into the feature matrix to serve as global temporal anchors. By injecting this irreversible, macroscopic deterioration trend, the feature engineering strategy forces the RF model to overcome the memoryless nature of conventional cross-sectional data processing, thereby locking onto the actual timeline of the ACB lifecycle. Following these baseline anchors, the EMCDI achieves the highest correlation score among all non-cumulative physical features, exceeding 0.75. This prominent ranking quantitatively validates the core physical assumption of this study: the existence of an interactive mechanical–jamming–electrical vicious cycle during the mid-to-late service stages of ACBs.

Furthermore, the moving average of the response delay $\mu_{\Delta T}$ and the transient contact resistance $R_{\mathrm{contact}}$ exhibit strong monotonic correlations. The robust correlations of $\mu_{\Delta T}$ demonstrates that the applied operational sliding window effectively filters out high-frequency transient thermal noise in industrial environments, successfully capturing the systematic mechanism jamming induced by the progressive drying of lubricating grease.

Conversely, mechanical and electrical features, such as the average opening angular velocity $\bar{\omega }$, contact initial separation angular velocity $\omega_{\mathrm{sep}}$, initial voltage slope of the opening arc $du_{\mathrm{arc}}/dt$, mechanical motion time $T_{\mathrm{motion}}$, single arc energy $W_{\mathrm{arc}}$, and single arcing time $T_{\mathrm{arc}}$, show moderate monotonic correlations. Although they carry explicit information regarding mechanism jamming and contact ablation, their monotonic deterioration trends are inevitably compromised by the inherent randomness of the switching phase angle $\phi_{\mathrm{switch}}$ and the transient current amplitudes during individual operations. Similarly, the moving variance of the response delay $\sigma_{\Delta T}^{2}$ and the TPEAI fall into this moderate range. The former primarily captures the stick-slip phenomenon during the late life stage, exhibiting abrupt mutations rather than continuous monotonic growth, while the latter reflects the unbalanced three-phase erosion that evolves with significant stochasticity rather than strict monotonicity.

Notably, mechanical geometric features that theoretically reflect physical contact thinning, such as the contact over-travel loss angle $\Delta \theta_{\mathrm{loss}}$ and the CSOCR, display unexpectedly lower correlation scores of approximately 0.4. This analytical deviation is primarily attributed to the mechanical impacts and dynamic rebounds of the energy-storage spring at the end of the switching action. These macroscopic structural vibrations severely pollute the steady-state readings of the angular sensor, injecting high-frequency mechanical noise that obscures the pure static contact ablation measurements.

Finally, variables that are completely independent of the aging process (e.g., the coil-to-arc response delay $\Delta T_{\mathrm{delay}}$), those constrained by rigid physical boundaries (e.g., the total contact angular stroke $\theta_{\mathrm{total}}$), and the purely random switching phase angle $\phi_{\mathrm{switch}}$ and arcing time $T_{\mathrm{arc}}$ rank lowest in the evaluation, with Spearman correlation coefficients below an empirical threshold of 0.3. These variables are subsequently excluded from the model inputs to prevent overfitting.

### 4.2. Principle of the Random Forest RUL Regression Model

A highly complex, nonlinear mapping relationship exists between the multidimensional features of ACBs and their remaining useful lifespan. The RF model, acting as a nonparametric regression method based on ensemble learning, exhibits strong anti-noise and anti-overfitting properties by constructing multiple independent decision trees and aggregating their prediction results. This architecture makes it particularly well-suited for handling the electromechanical coupling features extracted in this study.

A 15-dimensional feature vector X, having undergone the initial 50-operation health status benchmarking, is set as the model input. To address the practical industrial scenario where the total lifespan *N_f_* of an unseen target device is strictly unknown, this study defines the target output *Y_RUL_* of the regression model as the absolute RUL. It must be strictly emphasized that *N_f_* is an a posteriori value used exclusively for generating the ground-truth labels during the training phase. For the *k*th operation in the training set, its true label calculation is given by:

(14) $$Y_{RUL}\left(k\right)=N_{f}-k$$

where *N_f_* represents the total number of operations spanning the actual full life of the training device. For any unseen target device during inference, its specific *N_f_* remains entirely unknown. The RF model must implicitly infer the individualized degradation rate and the remaining life boundary solely relying on the multidimensional electro-mechanical feature matrix. Within this context, the cumulative operation count *k* and cumulative arc energy serve merely as historical temporal coordinates rather than deterministic target proxies. The training process of the RF model primarily involves the following core steps:

1. Bootstrap sampling: M sub-training sets, identical in size to the original training set, are randomly selected with replacement to generate M decision trees.
2. Random subspace splitting: During the feature splitting process at each node within a decision tree, rather than traversing all 15-dimensional features, a subset of features is randomly selected, and the optimal one is chosen for node splitting. This mechanism further reduces inter-tree correlation.
3. Integrated regression output: For a new test sample *X*, the RF model calculates the average of the M decision tree regression prediction results to output the final expected RUL value.

For a new device with an unknown total lifespan, the model receives real-time features *X*(*k*) that have been benchmarked based on its initial 50 operations. The average prediction results are regressed using the M decision trees, and the current absolute RUL expectation is directly outputted. The entire forward propagation process of the prediction requires no prior knowledge regarding the total lifespan of the newly deployed device.

(15) $${\hat{Y}}_{RUL}\left(k\right)=\frac{1}{M}\sum_{m=1}^{M}h_{m}\left(X\left(k\right),\Theta_{m}\right)$$

where *h_m_* is a single decision tree and $\Theta_{m}$ is the random variable vector determining the tree’s structure.

### 4.3. Model Training and Cross-Device Generalization Verification Strategy

To comprehensively evaluate the accuracy of the constructed RUL prediction model and the effectiveness of the phase-decoupled baseline normalization in eliminating manufacturing tolerances, this study designs a verification strategy that combines single device verification with cross device generalization transfer.

#### 4.3.1. Single-Device Verification

This study utilizes the lifecycle dataset of 1# ACB (referred to as ACB-1, which operated 2198 operations before failure) for foundational model training and testing. To ensure the mathematical validity of the model training and validation, a strict chronological partitioning strategy is adopted, dividing the sequential dataset into training, validation, and testing sets at a ratio of 7:1:2. This forward-chaining strategy prevents information leakage that would inevitably occur if strongly autocorrelated lifecycle observations were randomly distributed. On the one hand, it ensures that the training set contains sufficient samples for parameter learning, and on the other hand, it retains independent future chronological sets for hyperparameter selection and generalization evaluation, thereby minimizing evaluation bias.

#### 4.3.2. Cross-Device Transfer Verification

In practical industrial applications, the predictive model must be seamlessly deployable to newly installed devices that were not exposed during training phase. To validate this capability, this study conducts cross-device transfer experiments by extracting the full-lifecycle dataset of 2# ACB (referred to as ACB-2, lasting 2078 operations) to serve as the target domain test set. During the evaluation phase, prognostic errors were calculated against the a posteriori true life label of ACB-2. However, at the prediction input stage, strict simulation of real-world engineering deployment was enforced. Specifically, only the first 50 operations of ACB-2 were utilized for feature normalization benchmarking. The subsequent normalized real-time features were directly fed into the RF model (pre-trained solely on ACB-1) to achieve unsupervised target calibration, thoroughly verifying the model’s cross-device transferability.

#### 4.3.3. Model Evaluation Metrics

To scientifically quantify predictive performance and objectively benchmark the proposed framework against mainstream machine learning models, this study selects three standard statistical indicators, including the root mean square error (RMSE), mean absolute error (MAE), and coefficient of determination *R*^2^. Furthermore, an asymmetric penalty score (APS) is incorporated to evaluate safety-critical prediction behaviors. The APS is explicitly designed to penalize the industrial hazards of RUL overestimation, defined as follows:

(16) $$\mathrm{APS}=\sum_{i=1}^{n}s_{i}/n_{\mathrm{sample}}$$

(17) $$s_{i}=\left\{\begin{array}{cc} e^{-\frac{d_{i}}{13}}-1 & d_{i}<0\\ e^{\frac{d_{i}}{10}}-1 & d_{i}\geq 0 \end{array}\right.$$

where *n*_sample_ is the total number of test samples and the prediction error is defined as $d_{i}={\hat{y}}_{i}-y_{i}$. A lower APS indicates that the model’s predictive distribution is inherently biased toward conservatism. This indicator accurately reflects the true engineering value of diagnostic models by heavily prioritizing industrial safety and grid reliability over symmetric mathematical curve-fitting.

## 5. Results and Analysis

This section comprehensively validates the RF prediction model based on a full-lifecycle experimental dataset. The experiment first contrasts the predictive performance utilizing solely electrical features versus a full-dimensional feature set on ACB-1 to demonstrate the necessity of multisource synchronous feature fusion. Subsequently, cross-device transfer validation was conducted to verify the effectiveness of the feature standardization in overcoming manufacturing tolerances. Finally, a multidimensional quantitative comparison is conducted between the proposed RF model and mainstream machine learning models.

### 5.1. Single-Device Validation and Feature Ablation Study

**Figure 5:**
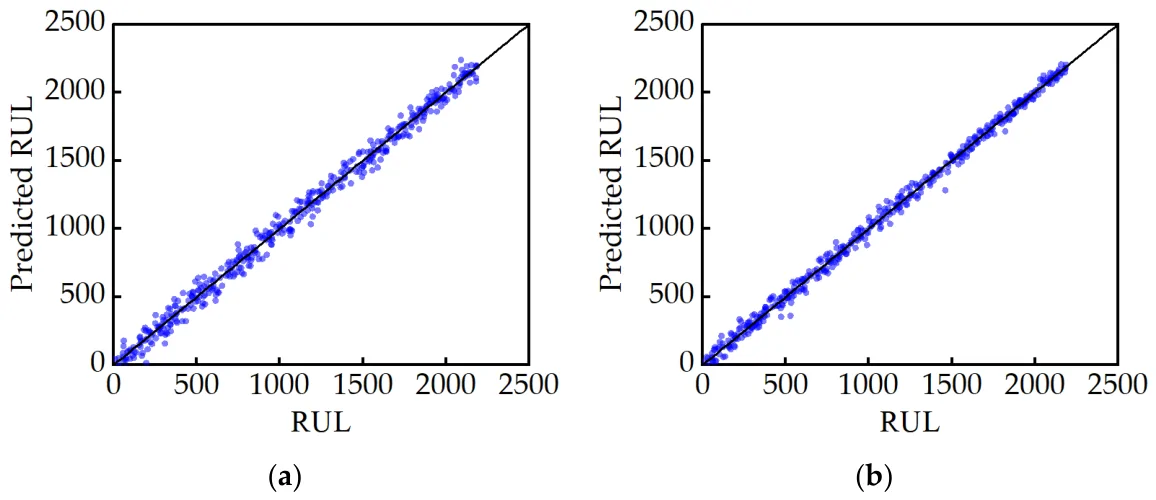
Results of the predicted RUL of ACB-1 using RF model. (**a**) Results with pure electrical feature set. (**b**) Results with full dimensional feature set.

To evaluate the predictive accuracy of the RF model and verify the necessity of multiphysics feature fusion, single-device closed-loop validation was conducted using the lifecycle dataset from ACB-1. The time-series samples were chronologically partitioned into training, validation, and testing sets according to a strict 7:1:2 ratio. Figure 5 contrasts the RUL prediction trajectories driven by two different feature spaces: Figure 5a utilizes purely electrical features with their accumulation, whereas Figure 5b incorporates the comprehensive feature matrix encompassing electrical, mechanical, electromechanical coupling features, and time-series accumulation. Table 2 provides the corresponding quantitative evaluation metrics, further stratified across three distinct lifecycle stages: the early stage (initial 400 operations), the middle stage (400 to 1800 operations), and the late stage (1800 operations to failure).

A detailed stage-by-stage analysis of Table 2 reveals the profound impact of introducing mechanical and electromechanical coupling features on the RUL prediction accuracy. During the early stage, the ACB operates in a relatively healthy, stable state with negligible structural wear or contact mass loss. In this aging phase, the predictive accuracy between the purely electrical model and the multiphysics model remains comparable, as the macroscopic physical degradation stimuli are too subtle to create a significant performance gap. However, as the device transitions into the middle stage, cumulative wear begins to manifest distinctly. As observed in Figure 5a, relying solely on electrical features during this protracted phase results in severe localized prediction fluctuations. This instability inherently stems from the highly stochastic nature of the arc plasma and the resulting volatile electrical signals. In stark contrast, Figure 5b and Table 2 demonstrate that incorporating mechanical kinematics drastically suppresses these oscillations. Mechanical deteriorations, such as progressive spring fatigue and microscopic transmission wear, evolve in a much more monotonic and deterministic manner compared to electrical variables. By integrating these physical anchors, the RF model significantly reduces the RMSE and MAE, providing a highly stable predictive trajectory throughout the middle lifecycle.

Furthermore, the indispensability of the comprehensive multiphysics feature set is most explicitly demonstrated during the late stage and rigorously captured by the APS in Table 2. While conventional symmetric metrics like RMSE and MAE quantify absolute numerical deviations, they critically fail to account for the physical directionality of prediction errors. In the physical context of low-voltage power distribution, overestimating the remaining life implies that an ACB, which is already suffering from severe contact over-travel loss or irreversible mechanism jamming, is erroneously deemed healthy and kept in service. The APS is specifically designed to explicitly penalize these hazardous late predictions far more heavily than conservative early predictions. As reported in Table 2, the pure electrical model fundamentally fails to capture the latent mechanical–jamming–electrical coupling effect in the late-life stage, resulting in massive trajectory divergence and dangerous life overestimations. Conversely, by accurately mapping the physical boundary conditions of mechanical wear, the multiphysics RF model yields a vastly optimized APS. This indicates that the model’s prediction distribution is inherently biased toward safety, guaranteeing that scheduled maintenance alerts are generated well before the critical threshold of mechanical or electrical failure is breached.

### 5.2. Cross-Device Transfer Validation and Extended Feature Ablation

In practical industrial applications, the total lifespan *N_f_* of an unseen target device is strictly unknown due to manufacturing tolerances and varying operational conditions. A robust RUL prediction framework must therefore rely on universally applicable physical degradation mechanisms rather than device-specific temporal anchors. To systematically validate the genuine transferability of the proposed method, the cross-device evaluation by training on ACB-1 and testing on ACB-2 is designed not only to assess the full framework but also to conduct an extended feature ablation study. This step-by-step ablation isolates the actual prognostic contribution of the physics-informed features from potential deterministic artifacts.

**Figure 6:**
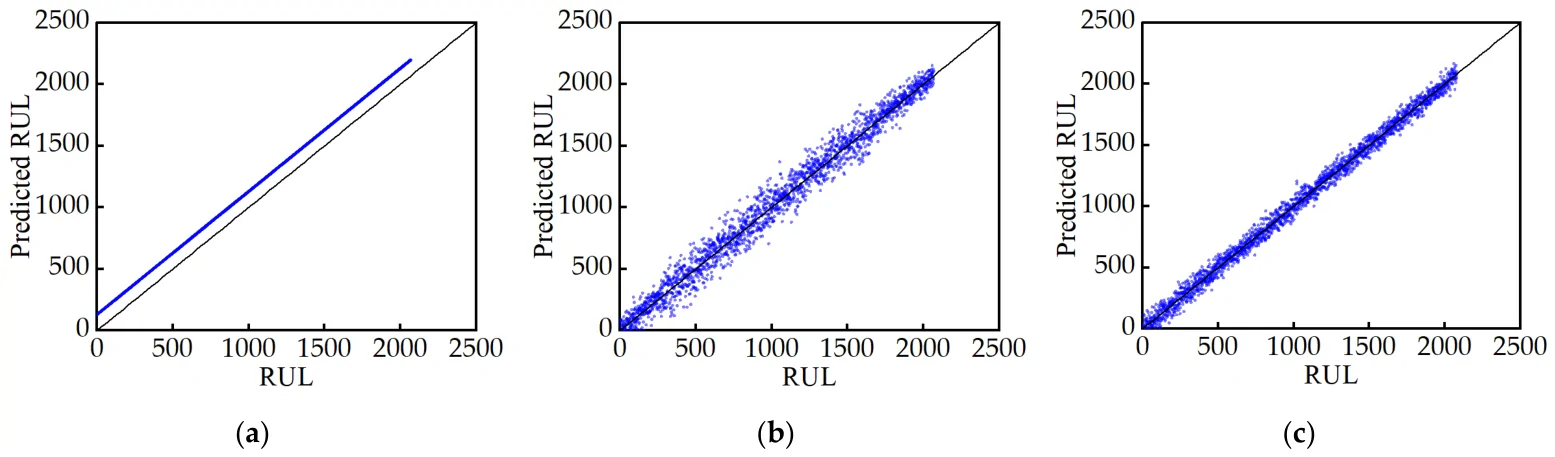
RUL prediction results of ACB-2 using the RF model trained on ACB-1 with different feature matrixes. (**a**) Operation-count-only. (**b**) Cumulative-feature-excluded. (**c**) Cumulative-feature-included.

This study first investigated an operation-count-only (OCO) baseline model. In data-driven prognostics, cumulative temporal variables often yield high mathematical correlation metrics during single-device training. However, their fundamental limitation becomes apparent during cross-device transfer. Having implicitly mapped the training device’s lifespan (ACB-1, *N_f_* = 2198), this baseline functions merely as a rigid, blind timer. As illustrated in Figure 6a, its predictions for ACB-2 exhibit a constant overestimation offset of approximately 120 operations. It entirely fails to adapt to the individualized degradation rate of the target device and is fundamentally incapable of detecting sudden physical anomalies in real-world scenarios.

To explicitly decouple the prediction from cumulative-features, this study subsequently evaluates a cumulative-feature-excluded (CFE) baseline. This model completely excludes all strongly cumulative features, forcing the RF model to rely exclusively on transient physical indicators captured during single switching events. As depicted in Figure 6b, the absence of cumulative features has a profound impact on the early-to-mid stage predictions. Without macroscopic temporal anchors (such as cumulative arc energy) to provide a global degradation baseline, the model loses its historical memory. Consequently, it becomes overly sensitive to the minor operational noise of a fundamentally healthy mechanism, resulting in localized fluctuations and a horizontal predictive trend rather than a smooth linear decline. However, during the late-life stage, this CFE model successfully locks onto the accelerated nonlinear degradation trajectory. Driven by abrupt mutations in physical indicators, the predicted RUL plunges to intercept the actual failure threshold, proving that the physical features contain genuine prognostic value independent of time.

Building upon these mechanistic insights, Figure 6c presents the performance of the proposed cumulative-feature-included (CFI) model. By synergizing the early-stage stability with late-stage physical sensitivity, the complete CFI model eliminates both the rigid overestimation offset of the OCO model and the early-stage oscillations of the CFE model, yielding a highly concentrated and accurate prediction distribution along the diagonal.

A quantitative comparative analysis further substantiates the impact of feature ablation. The OCO model achieves a high *R*^2^ of 0.9599, as well as high RMSE and MAE values of 120.22, and 120.22, respectively. According to the evaluation metrics presented in Table 3, the statistical penalty of lacking cumulative features is revealed in the CFE model. It yields a degraded full-lifecycle RMSE of 83.5837 and an MAE of 64.7116. The impact is prominent during the early and late stages, evidenced by a lower *R*^2^ of 0.5999 and an elevated RMSE of 73.0388. This mathematically underscores that relying solely on transient physical symptoms is insufficient to stably regress a linear life decay during the equipment’s healthy plateau. In contrast, the proposed CFI model integrates the time-series accumulation features to restore early-stage stability, improving the full-lifecycle RMSE to 48.1508 and the MAE to 38.5136, alongside an exceptional *R*^2^ of 0.9936.

Crucially, the cross-device validation confirms the successful transfer of the model’s safety-oriented prediction bias. Evaluated by the safety-critical APS, the proposed RF model registers a full-lifecycle score of 3.68 × 10^5^ on ACB-2, with the late-stage APS effectively controlled at 1.03 × 10^4^. This represents a significant safety improvement over the CFE model, which posted a higher late-stage APS of 6.29 × 10^4^. This demonstrates that the integrated multiphysics features consistently drive the model to issue conservative life estimations regardless of individual machine variances.

### 5.3. Comprehensive Comparison of Multiple Models

To comprehensively verify the superiority and engineering applicability of the proposed physics-informed RF framework, a horizontal comparison was conducted against six mainstream data-driven predictive algorithms. These benchmark algorithms encompass deep learning architectures (CNN, MLP, LSTM), a distance-based non-parametric model (KNN), and penalized linear regression models (Ridge, Lasso). All models were evaluated under a cross-device target calibration protocol on ACB-2, utilizing the identical phase-decoupled multiphysics feature matrix. The main parameter settings of the compared models are listed in Table 4.

**Figure 7:**
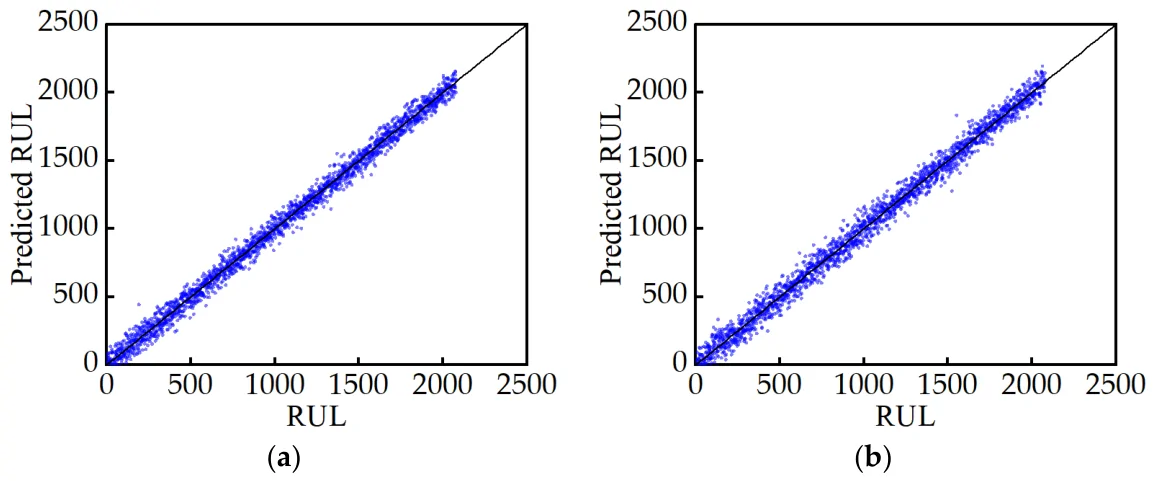

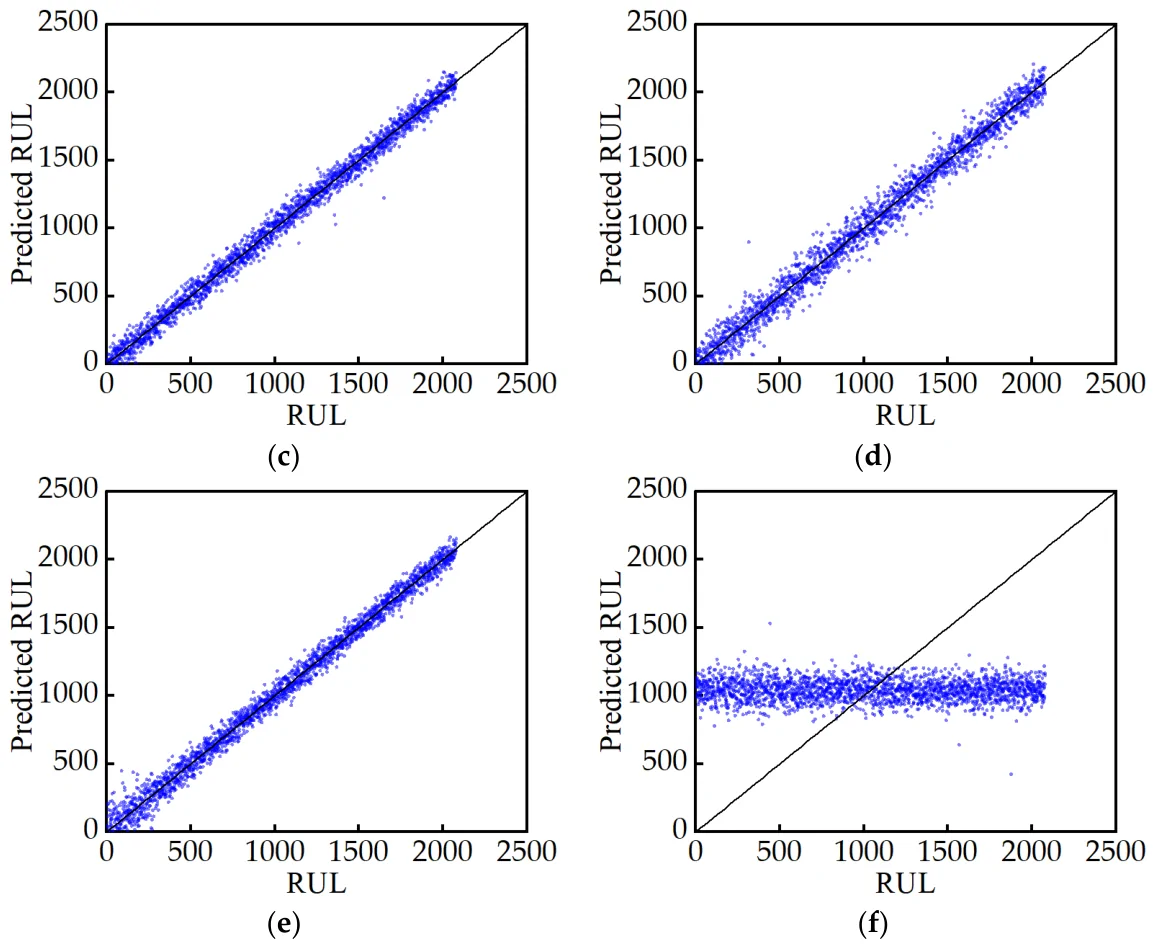
Comparison of predicted RUL trajectories for ACB-2 using different models. (**a**) CNN. (**b**) MLP. (**c**) LSTM. (**d**) KNN. (**e**) Ridge. (**f**) Lasso.

The corresponding prediction scatter distributions and quantitative evaluation metrics are detailed in Figure 7 and Table 5. From the perspective of predictive accuracy and nonlinear mapping capability, the proposed RF model consistently outperforms all algorithms, achieving the lowest RMSE of 48.1508, the lowest MAE of 38.5136, and the highest *R*^2^ of 0.9936. In sharp contrast, the penalized linear regression models exhibit significant limitations in capturing the complex degradation mechanisms of ACBs. While the Ridge regression maintains a baseline accuracy (*R*^2^ of 0.9892), it struggles to map the severe nonlinear mutations driven by the electromechanical vicious cycle near the end of life. The Lasso model entirely fails to converge. Similarly, the distance-based KNN model suffers from the curse of dimensionality within the 15-dimensional multiphysics feature space, resulting in elevated error bounds.

The engineering value of the models is fundamentally differentiated by the safety-critical APS. In the context of low-voltage distribution networks, conventional symmetric metrics (RMSE, MAE) mask risks by overestimating the RUL at the late-life stage. If a severely degraded ACB is falsely predicted to be healthy, it may fail to interrupt a short-circuit fault, precipitating thermal runaway and widespread outages. The quantitative results reveal that while deep learning models maintain acceptable RMSE levels, their APS values surge exponentially (e.g., the LSTM model’s full-lifecycle APS reaches a magnitude of 10^11^). This exposes a dangerous mathematical tendency of neural networks to generate overestimations near the critical failure boundary. The proposed RF framework registers a minimized and stable APS of 3.68 × 10^5^, quantitatively demonstrating its conservative and risk-averse predictive tendency. By accurately responding to precursor electromechanical anomalies, the RF model ensures that highly reliable maintenance alerts are triggered well in advance of ultimate structural failure, satisfying the reliability demands of condition-based maintenance in smart grids.

### 5.4. Model Interpretability and Physics-Informed Validation

While the preceding sections demonstrate the model’s predictive accuracy and cross-device transferability, unpacking its underlying decision-making process is essential for building trust in practical engineering applications. To understand how the RF model arrives at its prognostic conclusions, this study evaluated the out-of-bag permutation importance. By measuring the increase in mean squared error (MSE) when a specific feature is randomly shuffled, this technique explicitly quantifies which indicators the model relies on most heavily during actual inference.

**Figure 8:**
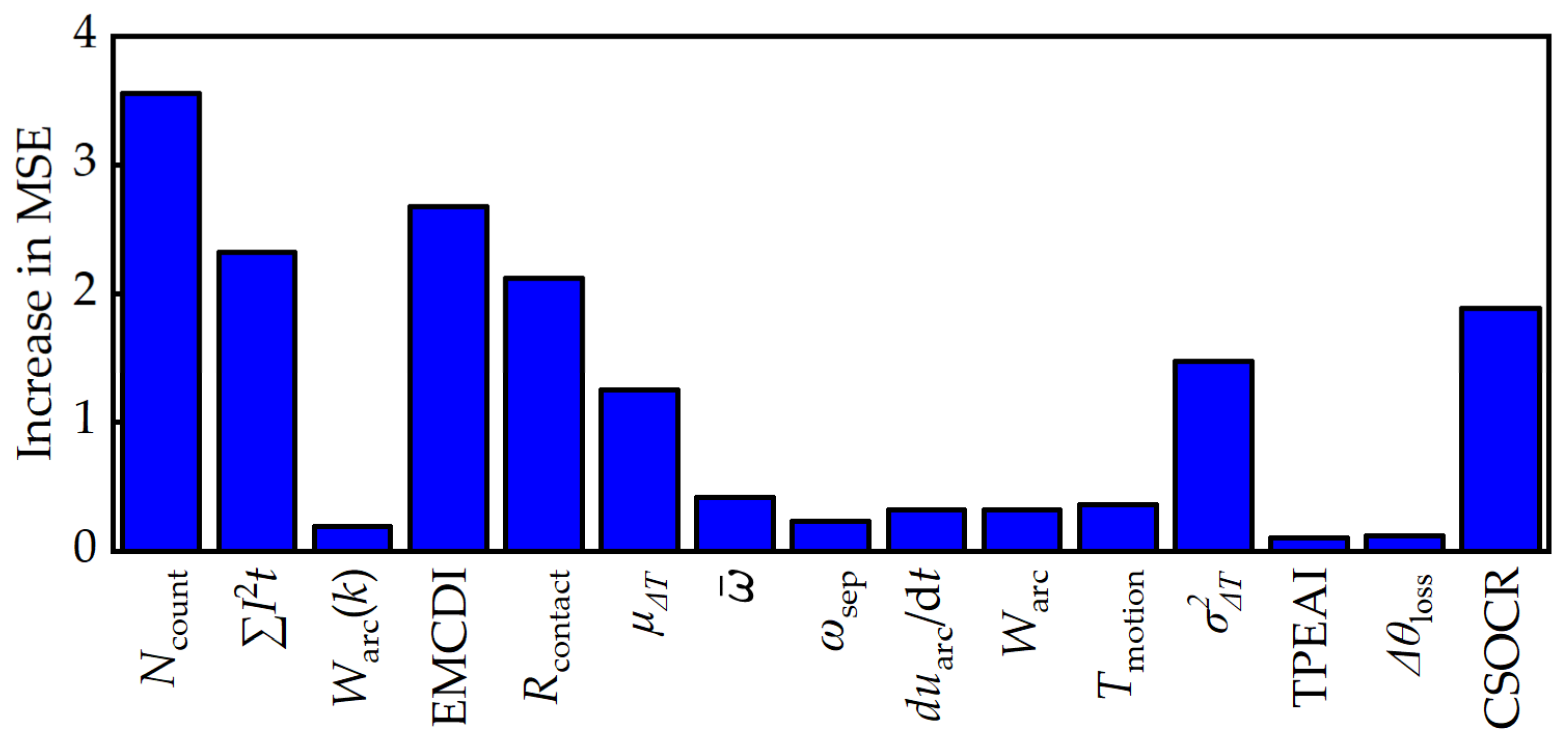
Permutation importance of multi-dimensional features.

As presented in Figure 8, the cumulative operation count $N_{\mathrm{count}}$ serves as the primary temporal anchor, establishing the macroscopic timeline of the aging lifecycle. However, the most compelling finding lies in how the model prioritizes the physical indicators. The proposed EMCDI emerges as the most vital physical feature (ranking second overall, even surpassing the cumulative arc energy $\sum I^{2}t$). This indicates that the model has autonomously learned to focus on the mechanical–jamming–electrical interactive deterioration, rather than merely tracking basic thermal accumulation.

Furthermore, the model exhibits a strong reliance on features characterizing contact surface degradation, specifically the transient contact resistance $R_{\mathrm{contact}}$ and the CSOCR. An interesting contrast emerges when comparing this model-level interpretation with the initial data-level Spearman assessment in Figure 4. Features such as the CSOCR and the moving variance of response delay $\sigma_{\Delta T}^{2}$ showed only moderate monotonic correlations, primarily because they remain largely flat during the extended healthy plateau of the device. Yet, they display prominent importance scores in the RF model. This reveals that the algorithm effectively utilizes its nonlinear tree structure to capture late-stage physical mutations, such as mechanical stick-slip phenomena or over-travel exhaustion, which are critical precursors to ultimate failure.

Conversely, instantaneous single-operation features (e.g., single arc energy $W_{\mathrm{arc}}\left(k\right)$, separation velocity $\omega_{\mathrm{sep}}$) show minimal impact on the MSE. This further confirms that the model is robust; it ignores high-frequency stochastic noise caused by fluctuating switching phase angles, focusing instead on trend indicators. Overall, this internal feature weighting confirms that the data-driven framework is a physics-aware system that prioritizes actual electromechanical failure symptoms to ensure reliable RUL predictions.

## 6. Conclusions

This study proposes a physics-informed, explainable RUL prediction framework for ACBs to overcome the engineering bottlenecks of physical fragmentation and small-sample generalization. Through full-lifecycle electromechanical synchronous monitoring, the complex physical degradation mechanisms of ACBs were successfully decoded and integrated into a robust data-driven model. The primary conclusions are drawn as follows:

1. Physics-informed feature engineering: A comprehensive feature matrix was constructed, systematically integrating electrical, mechanical, electromechanical coupling, and time-series accumulation features. The introduction of novel indicators, specifically the electromechanical coupled degradation index (EMCDI) and the contact spring over-travel consumption rate (CSOCR), effectively captures and quantifies the complex interactive deterioration cycle between arc ablation and mechanism jamming.
2. Model interpretability and physics-aware decision-making: By evaluating the out-of-bag permutation importance, this study elucidates the internal decision-making mechanism of the RF model. The model-level analysis reveals that the regressor assigns paramount weights to the proposed EMCDI and the transient contact resistance. This confirms that the framework captures the mechanical–jamming–electrical interactive deterioration rather than relying on spurious statistical correlations.
3. Cross-device transferability: To eliminate the interference of initial manufacturing tolerances and the randomness of breaking phase angles, a phase-decoupled baseline normalization strategy was implemented. By extracting health benchmarks from only the first 50 operations of a target device, the proposed model successfully transferred to a new, unseen device. It achieved an exceptional full-lifecycle *R*^2^ of 0.9936, an RMSE of 48.1508, and an MAE of 38.5136.
4. Superiority in small-sample nonlinear mapping and safety-oriented reliability: Under a cross-device target calibration protocol trained on a limited physical dataset, the RF model consistently outperformed mainstream deep learning algorithms (CNN, MLP, LSTM) and penalized linear regression models (Ridge, Lasso). The proposed framework demonstrated an exceptional capability to map the severe nonlinear mutations that occur near the end of life. Validated by the safety-critical asymmetric penalty score (APS), the proposed RF framework consistently achieves a conservative, risk-averse RUL prediction. This ensures that maintenance alerts are reliably triggered before ultimate structural failure, satisfying the safety requirements of condition-based maintenance in smart grids.

While the proposed framework demonstrates robust cross-device transferability, the experimental sample size remains a limitation due to the prohibitive time and economic costs of full-lifecycle testing. The current experimental validation is restricted to two ACBs with identical specifications (400 V AC, 2000 A). Future research will deploy this prediction framework across a larger-scale industrial switchgear cluster to further verify its statistical reliability.

## Figures and Tables

**Table 1:** Summary of extracted multi-physics features.

Category	Feature Name	Abbreviation	Unit
Electrical	Arc energy	$W_{\mathrm{arc}}$	J
	Arcing time	$T_{\mathrm{arc}}$	ms
	Switching phase angle	$\phi_{\mathrm{switch}}$	rad
	Initial voltage slope of the opening arc	$du_{\mathrm{arc}}/dt$	V/ms
	Transient contact resistance	$R_{\mathrm{contact}}$	μΩ
	Coil-to-arc response delay	$\Delta T_{\mathrm{delay}}$	ms
	Three-phase energy asymmetry index	TPEAI	-
Mechanical	Average opening angular velocity	$\bar{\omega }$	rad/s
	Contact initial separation angular velocity	$\omega_{\mathrm{sep}}$	rad/s
	Total contact angular stroke	$\theta_{\mathrm{total}}$	rad
	Contact over-travel loss angle	$\Delta \theta_{\mathrm{loss}}$	rad
	Mechanical motion time	$T_{\mathrm{motion}}$	ms
Electromechanical coupling	Electromechanical coupled degradation index	EMCDI	J⋅s/rad
	Contact spring over-travel consumption rate	CSOCR	rad/J
Time-series accumulation	Cumulative operation count	$N_{\mathrm{count}}$	-
	Cumulative arc energy	$W_{\mathrm{arc}}\left(k\right)$	J
	Cumulative cut-off joule integral	$\sum I^{2}t$	$A^{2}\cdot s$
	Moving average of response delay	$\mu_{\Delta T}$	ms
	Moving variance of response delay	$\sigma_{\Delta T}^{2}$	ms^2^

**Table 2:** Comparison of evaluation metrics for ACB-1 at different life stages.

Life Stage	Pure Electrical Feature Set				Full Dimensional Feature Set			
	RMSE	MAE	*R* ^2^	APS	RMSE	MAE	*R* ^2^	APS
Full	56.0256	44.4455	0.9923	1.93 × 10^4^	38.9283	29.9045	0.9963	3.09 × 10^3^
Early	58.2100	45.2235	0.7170	1.95 × 10^4^	39.6355	32.5765	0.8685	1.75 × 10^3^
Middle	57.7561	46.9780	0.9800	1.25 × 10^4^	41.3091	31.5092	0.9898	4.41 × 10^3^
Late	47.1667	35.2073	0.8222	4.18 × 10^4^	28.6678	21.7927	0.9343	8.64 × 10^1^

**Table 3:** Evaluation metrics of ACB-2 at different life stages.

Life Stage	RMSE	MAE	*R* ^2^	APS
Full	48.1508	38.5136	0.9936	3.68 × 10^5^
Early	44.8241	36.1161	0.6880	1.98 × 10^3^
Middle	47.1989	37.6536	0.9864	1.12 × 10^5^
Late	53.3957	43.1897	0.7862	1.03 × 10^4^

**Table 4:** Main parameter settings of the compared models.

Model	Key Parameter	Setting
RF	n_estimators/max_depth	100/10
CNN	conv filters/kernel size	16,32/3
MLP	hidden units	128-64-32
LSTM	hidden units	64
KNN	K	5
Ridge	α	1.0
Lasso	α	0.01
Unified training strategy	batch size/epochs/optimizer/learning rate/loss/patience/L2/dropout	32/100/Adam/0.001/MSE/10/0.001/0.2

**Table 5:** Comparison of evaluation metrics for ACB-2 using benchmark models across different life stages.

Life Stage	Statistical Indicators	RF	CNN	MLP	LSTM	KNN	Ridge	Lasso
Full	RMSE	48.1508	51.9742	59.4060	57.5598	82.0714	62.2058	606.8985
	MAE	38.5136	41.2216	46.9707	45.4169	63.7711	48.1344	525.0041
	*R* ^2^	0.9936	0.9925	0.9902	0.9908	0.9813	0.9892	−0.0236
	APS	3.68 × 10^5^	2.27 × 10^8^	3.70 × 10^9^	3.14 × 10^11^	5.75 × 10^22^	1.32 × 10^13^	5.83 × 10^47^
Early	RMSE	44.8241	48.9161	52.3987	52.0998	74.9453	51.6648	903.0088
	MAE	36.1161	39.8030	41.5953	41.5391	61.2860	41.8745	895.7176
	*R* ^2^	0.6880	0.6285	0.5737	0.5785	0.1279	0.5855	−125.6139
	APS	1.98 × 10^3^	3.01 × 10^3^	1.44 × 10^4^	1.67 × 10^5^	2.56 × 10^6^	2.74 × 10^3^	8.43 × 10^45^
Middle	RMSE	47.1989	51.0663	60.0333	58.7903	80.9807	53.7364	417.6959
	MAE	37.6536	40.0694	47.1394	46.1747	62.8213	43.1624	359.7890
	*R* ^2^	0.9864	0.9840	0.9779	0.9788	0.9598	0.9823	−0.0682
	APS	1.12 × 10^5^	8.37 × 10^4^	1.16 × 10^9^	9.87 × 10^10^	3.49 × 10^9^	7.47 × 10^4^	1.71 × 10^44^
Late	RMSE	53.3957	56.9491	61.7361	56.8171	90.1988	90.2257	857.9556
	MAE	43.1897	46.2406	50.1163	45.4595	68.8226	69.8870	845.6110
	*R* ^2^	0.7862	0.7568	0.7141	0.7579	0.3898	0.3894	−54.2069
	APS	1.03 × 10^4^	2.50 × 10^8^	1.86 × 10^6^	7.76 × 10^4^	6.33 × 10^22^	1.46 × 10^13^	6.35 × 10^47^

## Data Availability

Data is contained within the article.
